# The Effects of Mandatory Prescribing of Thiazides for Newly Treated, Uncomplicated Hypertension: Interrupted Time-Series Analysis

**DOI:** 10.1371/journal.pmed.0040232

**Published:** 2007-07-10

**Authors:** Atle Fretheim, Kari Håvelsrud, Graeme MacLennan, Doris Tove Kristoffersen, Andrew D Oxman

**Affiliations:** 1 Norwegian Knowledge Centre for the Health Services, Oslo, Norway; 2 Health Services Research Unit, University of Aberdeen, Scotland, United Kingdom; George Institute for International Health, Australia

## Abstract

**Background:**

The purpose of our study was to evaluate the effects of a new reimbursement rule for antihypertensive medication that made thiazides mandatory first-line drugs for newly treated, uncomplicated hypertension. The objective of the new regulation was to reduce drug expenditures.

**Methods and Findings:**

We conducted an interrupted time-series analysis on prescribing data before and after the new reimbursement rule for antihypertensive medication was put into effect. All patients started on antihypertensive medication in 61 general practices in Norway were included in the analysis. The new rule was put forward by the Ministry of Health and was approved by parliament. Adherence to the rule was monitored only minimally, and there were no penalties for non-adherence. Our primary outcome was the proportion of thiazide prescriptions among all prescriptions made for persons started on antihypertensive medication. Secondary outcomes included the proportion of patients who, within 4 mo, reached recommended blood-pressure goals and the proportion of patients who, within 4 mo, were not started on a second antihypertensive drug. We also compared drug costs before and after the intervention. During the baseline period, 10% of patients started on antihypertensive medication were given a thiazide prescription. This proportion rose steadily during the transition period, after which it remained stable at 25%. For other outcomes, no statistically significant differences were demonstrated. Achievement of treatment goals was slightly higher (56.6% versus 58.4%) after the new rule was introduced, and the prescribing of a second drug was slightly lower (24.0% versus 21.8%). Drug costs were reduced by an estimated Norwegian kroner 4.8 million (€0.58 million, US$0.72 million) in the first year, which is equivalent to Norwegian kroner 1.06 per inhabitant (€0.13, US$0.16).

**Conclusions:**

Prescribing of thiazides in Norway for uncomplicated hypertension more than doubled after a reimbursement rule requiring the use of thiazides as the first-choice therapy was put into effect. However, the resulting savings on drug expenditures were modest. There were no significant changes in the achievement of treatment goals or in the prescribing of a second antihypertensive drug.

## Abstract 2

Atle Fretheim and colleagues found that the prescribing of thiazides in Norway for uncomplicated hypertension more than doubled after a rule requiring their use as first-choice therapy was put into effect.

## Editors' Summary

**Background.:**

High blood pressure (hypertension) is a common medical condition, especially among elderly people. It has no obvious symptoms but can lead to heart attacks, heart failure, strokes, or kidney failure. It is diagnosed by measuring blood pressure—the force that blood moving around the body exerts on the inside of arteries (large blood vessels). Many factors affect blood pressure (which depends on the amount of blood being pumped round the body and on the size and condition of the arteries), but overweight people and individuals who eat fatty or salty food are at high risk of developing hypertension. Mild hypertension can often be corrected by making lifestyle changes, but many patients also take one or more antihypertensive agents. These include thiazide diuretics and several types of non-thiazide drugs, many of which reduce heart rate or contractility and/or dilate blood vessels.

**Why Was This Study Done?:**

Antihypertensive agents are a major part of national drug expenditure in developed countries, where as many as one person in ten is treated for hypertension. The different classes of drugs are all effective, but their cost varies widely. Thiazides, for example, are a tenth of the price of many non-thiazide drugs. In Norway, the low use of thiazides recently led the government to impose a new reimbursement rule aimed at reducing public expenditure on antihypertensive drugs. Since March 2004, family doctors have been reimbursed for drug costs only if they prescribe thiazides as first-line therapy for uncomplicated hypertension, unless there are medical reasons for selecting other drugs. Adherence to the rule has not been monitored, and there is no penalty for non-adherence, so has this intervention changed prescribing practices? To find out, the researchers in this study analyzed Norwegian prescribing data before and after the new rule came into effect.

**What Did the Researchers Do and Find?:**

The researchers analyzed the monthly antihypertensive drug–prescribing records of 61 practices around Oslo, Norway, between January 2003 and November 2003 (pre-intervention period), between December 2003 and February 2004 (transition period), and between March 2004 and January 2005 (post-intervention period). This type of study is called an “interrupted time series”. During the pre-intervention period, one in ten patients starting antihypertensive medication was prescribed a thiazide drug. This proportion gradually increased during the transition period before stabilizing at one in four patients throughout the post-intervention period. A slightly higher proportion of patients reached their recommended blood-pressure goal after the rule was introduced than before, and a slightly lower proportion needed to switch to a second drug class, but both these small differences may have been due to chance. Finally, the researchers estimated that the observed change in prescribing practices reduced drug costs per Norwegian by US$0.16 (€0.13) in the first year.

**What Do These Findings Mean?:**

Past attempts to change antihypertensive-prescribing practices by trying to influence family doctors (for example, through education) have largely failed. By contrast, these findings suggest that imposing a change on them (in this case, by introducing a new reimbursement rule) can be effective (at least over the short term and in the practices included in the study), even when compliance with the change is not monitored nor noncompliance penalized. However, despite a large shift towards prescribing thiazides, three-quarters of patients were still prescribed non-thiazide drugs (possibly because of doubts about the efficacy of thiazides as first-line drugs), which emphasizes how hard it is to change doctors' prescribing habits. Further studies are needed to investigate whether the approach examined in this study can effectively contain the costs of antihypertensive drugs (and of drugs used for other common medical conditions) in the long term and in other settings. Also, because the estimated reduction in drug costs produced by the intervention was relatively modest (although likely to increase over time as more patients start on thiazides), other ways to change prescribing practices and produce savings in national drug expenditures should be investigated.

**Additional Information.:**

Please access these Web sites via the online version of this summary at http://dx.doi.org/10.1371/journal.pmed.0040232.

- MedlinePlus encyclopedia page on hypertension (in English and Spanish)
- UK National Institute for Health and Clinical Excellence information on hypertension for patients, carers, and professionals
- American Heart Association information for patients on high blood pressure
- An open-access research article describing the potential savings of using thiazides as the first-choice antihypertensive drug
- A previous study  in Norway, published in *PLoS Medicine,* examined what happened when doctors were actively encouraged to make more use of thiazides. There was also an economic evaluation  of what this achieved

## Introduction

Antihypertensive medication is one of the major drug expenditures for the national drug-reimbursement scheme in Norway, adding up to around Norwegian kroner (NOK) 1,500 million per year (€180 million, US$220 million) [1], i.e., NOK330 per inhabitant (€40, US$49). This represents 10% of all registered drug sales. The use of antihypertensive drugs has been increasing steadily over many years. However, their proportional contribution to the overall drug expenditure in Norway has decreased slightly (Table 1). An estimated 6%–10% of the Norwegian population is being treated for hypertension [2].

**Table 1:**
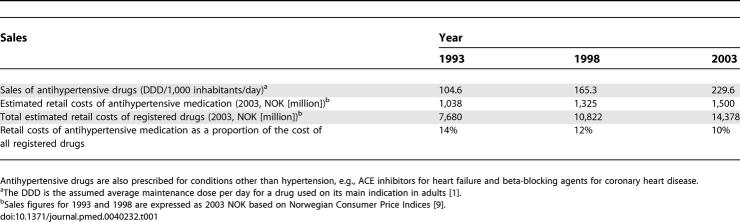
Sales of Antihypertensive Drugs in Norway 1993–2003

Several drugs are available for the treatment of hypertension and, in terms of effectiveness, there is little or no difference between them. From a public health and economic perspective, the major difference between the drugs is probably the price. The cost of many non-thiazide drugs is 10-fold that of thiazides [2].

Norway is one country where the use of thiazides is particularly low and the use of expensive alternatives is high [2], and this has been an issue of concern for the Ministry of Health as well as for Norwegian parliamentarians [3].

In late 2003, the Norwegian parliament passed a regulation aimed at reducing public expenditure on antihypertensive drugs. Choosing a thiazide as the first-choice therapy for uncomplicated hypertension became a prerequisite for the reimbursement of drug costs, unless there were medical reasons for selecting other drugs. The new reimbursement rule was officially presented on 15 January 2004 and was put into effect on 1 March 2004.

Substantial savings were expected to result from the new reimbursement rule. However, factors other than the anticipated increase in thiazide use may affect expenditures. For instance, some critics believe that thiazides are less effective in controlling blood pressure and that the need for add-on drugs will outweigh the economic gains.

The effects of health-policy interventions are often poorly evaluated. Owing to pressures to implement health-policy interventions quickly and widely, it is not always feasible to conduct rigorous evaluations, such as a randomised controlled trial. Sometimes the nature of the intervention is such that a control group is difficult to establish, e.g., owing to mass-media campaigns.

**Figure 1:**
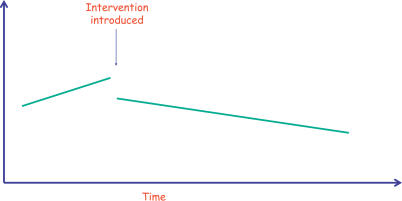
An ITS is a Quasi-Experimental Design that Can Be Used to Evaluate an Intervention when a True Randomised Controlled Experiment Is Not Feasible In this study, the intervention was a policy change that was being delivered to all physicians in Norway and, because of this, no control was available. An ITS design strengthens a before-and-after design by taking repeated measurements of the outcome over time, both pre- and post-intervention. Using appropriate statistical methods, it is then possible to estimate the effect of an intervention given the underlying trend of the data, and taking into account effects such as seasonality or serial correlation. There are a variety of possible intervention effects in an ITS experiment, for example where the intervention has had an effect on the slope and level of the outcome.

If a randomised evaluation is not possible, a before-and-after study may be an alternative. However, if measurements are only made once before and once after the intervention, the findings may be misleading. For instance, if changes in prescribing of a drug were observed before and after an intervention, this could simply reflect an ongoing trend that is independent of the intervention. Another explanation for an observed change could be other influences that coincide with the intervention. One way of increasing the robustness of a before-and-after analysis is to collect several data points before and after the intervention—an interrupted time-series (ITS) analysis (see Figure 1) [4].

The primary objectives of this study were to measure changes in prescribing of thiazides for newly treated, uncomplicated hypertension following the introduction of the new reimbursement rule. Also, we explored possible changes in proportions of people achieving recommended blood-pressure targets and the need for add-on drugs or switching of medication.

## Methods

We conducted an ITS analysis, with 11 measurements at monthly intervals before and after the intervention. We incorporated a transition period from December 2003 to February 2004. The study protocol (Text S1) is available on our Web site (http://www.nokc.no).

### Participants

We included general practices in and around Oslo, Norway. The sample was drawn from three different practice populations: (1) practices randomised to the intervention group in a recently completed trial of a multifaceted intervention aimed at improving adherence to clinical practice guidelines for the pharmacological treatment of hypertension, where prescribing of thiazides was an outcome measure [5]; (2) practices from the control group in the same trial; and (3) practices that had not participated in the trial.

### Intervention

A new rule from the Norwegian Medicines Agency made thiazides the only reimbursed drug class for uncomplicated hypertension, i.e., in patients who had no hypertensive organ damage, and no gout, reduced glucose tolerance, or untreated diabetes. Non-thiazide drugs may be reimbursed provided that a medical reason is given in the medical record.

Information about the new rule was circulated to all physicians through the January 2004 edition of the bulletin of the Norwegian Medicines Agency [6]. The rule was also widely debated in the media [7].

No specific sanctions were announced, but it was stated that the National Insurance Administration, which manages the reimbursement of drugs, was about to increase its monitoring activities of adherence to reimbursement rules, generally. In theory, a physician who does not adhere to the reimbursement rule may lose the right to issue prescriptions that are to be covered by the national insurance scheme. However, this has never happened in practice. During the first year after introduction of the new rule, there was a minimal amount of monitoring.

The intervention was not completely independent of other changes that have taken place over time. A debate on the choice of medication for hypertension has been going on for several years within the medical community, and also in the public domain. For instance, an economic analysis on potential savings if thiazides were used as the first-line medication [2] made the headlines in Norway in September 2003 [8]. Also, the new regulation prompted a debate, widely covered in the media, where opinion leaders and other stakeholders had strongly divergent views regarding the use of thiazides for the treatment of hypertension [7]. This may be regarded as a direct consequence of the intervention and not a source of bias. The 3-mo transition period incorporated in our analysis eliminates any short-term effects of this debate on our results. However, it is not possible to determine what, if any, effect the debate had on raising awareness of the policy and, thereby, adherence.

### Data Collection

We collected data from electronic medical records held at the practices involved in the study. The data included all prescribed antihypertensive drugs (Anatomic Therapeutic Chemical [ATC] groups C02, C03A, C03E, C07, C08, and C09) from 3 y before and 1 y after the reimbursement rule was implemented.

For all patients who had been given a prescription for an antihypertensive drug, we also collected information on cardiovascular diagnoses (International Classification of Primary Care [ICPC] codes K74–80, K84, K89–92, and K99) and prescribing of cardiovascular drugs (ATC group C01). This was to identify patients who might have been taking the drugs for reasons other than hypertension. We also extracted blood-pressure measurements, and only patients with a recorded measurement above 140/90 mm Hg were included in the main analyses.

Because antihypertensive drugs are also prescribed to treat migraine or hyperthyroidism, we identified patients with these diagnoses (ICPC codes N89 and T85) and prescriptions for drugs to treat these conditions (ATC codes N02C and H03B). If these diagnoses or prescriptions were recorded, the patient was excluded from the analysis.

The new reimbursement rule included only patients treated for uncomplicated hypertension, defined in this case as not having target-organ damage, gout, decreased glucose tolerance, or untreated diabetes. In order to identify patients with these conditions, we extracted information on relevant diagnoses (K87, T92, T89, and T90) and prescriptions (M04 and A10). We did not attempt to identify patients with decreased glucose tolerance, as we did not believe that the available data would enable us to do this in a reliable way. Patients with a recorded glucose measurement of ≥7 mmol/l and no recorded prescriptions for antidiabetic medication or other diabetes aids were considered as having untreated diabetes, and were thus excluded from the main analyses.

We have no reason to believe that the intervention affected data collection, which was the same for all included data.

### Outcomes

In the protocol, we defined outcomes in terms of undesired events (e.g., the proportion of non-thiazide prescribing). We decided to invert the outcomes and to report desired events instead, in the belief that this is easier to understand. We also initially planned in the protocol to report two indicators of inappropriate prescribing as one composite outcome measure. However, we subsequently decided to report them separately as we believed that mixing the two measures could be confusing for the reader, particularly since many patients had both outcomes.

The primary outcome was the proportion of thiazide prescriptions among all prescriptions made for persons started on antihypertensive medication. Drugs belonging to ATC groups C03A and C03E were counted as thiazides. Patients were considered untreated if there was no record of a prescription for an antihypertensive drug in the previous 22 mo.

Secondary outcomes could be classified into five groups: (1) subgroup analysis of primary outcome where all patients with diabetes were excluded (patients with untreated diabetes were excluded from the main analysis); (2) the proportion of patients who, within 4 mo, reached recommended blood-pressure goals; (3) the proportion of patients who, within 4 mo, were not started on a second antihypertensive drug; (4) the proportion of patients with heart failure being prescribed an angiotensin-converting enzyme (ACE) inhibitor (ATC groups C09A and C09B) within 3 mo of receiving the diagnosis; and (5) the proportion of patients with coronary heart disease being prescribed a beta-blocking agent (ATC group C07) within 3 mo of receiving the diagnosis (proxy measures for inappropriate prescribing).

For the secondary outcomes, the intervention period was shortened to 8 mo to allow for sufficient follow-up for all included patients.

We also conducted a subgroup analysis, comparing the main outcome for the three groups of practices that participated in the study.

These are objective outcomes based on data extracted electronically from medical records that required little or no interpretation; no blinding or reliability testing of outcome evaluation was therefore considered necessary.

### Economic Evaluation

We conducted a post-hoc estimation of the savings on drug expenditures from the perspective of the health system, i.e., those who pay for health care. In Norway, antihypertensive medication is largely paid for by the government. We estimated the savings by extrapolating the drug expenditures in the pre-intervention and the post-intervention periods to include all practices in Norway for a full year. For each antihypertensive prescription for someone started on antihypertensive medication during the pre-intervention period, we applied drug prices as of 15 November 2003 from the 2004 edition of the Norwegian Pharmaceutical Product Compendium (“Felleskatalogen”) and, for the post-intervention period, we applied prices from the 2005 edition (prices as of 1 December 2004). Thus, the economic evaluation was a simple before-and-after analysis, not an ITS analysis. We excluded value-added tax and adjusted for the general price increase from 2003 to 2004 using consumer price indices from Statistics Norway [9]. All figures are expressed in 2004 NOK.

### Ethics

We asked for consent from all the participating practices (Text S2). Physicians and patients were not identifiable from the collected data. No ethical approval was needed. The Norwegian Social Science Data Services approved the handling of data.

### Sample Size and Statistics

In the planning of a previous randomised trial [5], we had estimated a needed sample size of around 140 practices in order to demonstrate a 25% relative reduction in outcome measures between the control and intervention groups, with a power of 80% and a statistical significance level of 5%. This was based on the assumption that none of the outcomes of interest would be less than 50% in the control group, that the average number of patients included per practice would be around ten patients per outcome measure, and that the intracluster correlation coefficient would be around 0.2. In the current study, the recruited practices would serve as their own control, and we knew that the baseline level of prescribing non-thiazides greatly exceeded 50%. Thus, assuming no serial correlation or trend over time, we estimated a need for less than half the sample size compared with the previous study, i.e., around 60 practices.

Data were analysed using generalised estimating equations (GEE) in Stata (version 9.2) using the xtgee procedure and logit link function, adjusting for clustering and potential serial correlation at the practice level. Data were too sparse to account for clustering and serial correlation at the physician level. An exchangeable correlation structure was used. Parameters and effect sizes are either presented as odds ratios with 95% confidence intervals (CIs) or are converted to percentages with 95% CIs for ease of interpretation. All outcomes were re-analysed, removing the transition phase to corroborate the results of the primary analyses.

## Results

We invited 106 practices to participate, of which 64 agreed and provided us with written consent. Three practices were excluded owing to technical difficulties, and thus our analyses are based on data from 61 practices. Among these, 19 practices were from the intervention group in a trial that we had recently conducted [5], 21 practices were from the control group in the same trial, and 21 practices had not participated in the trial. The number of patients started on antihypertensive medication was similar in the two time periods (1,628 patients before and 1,580 patients after the rule was introduced). The aggregated results for before and after the intervention, as well as during the transition period, are shown in Table 2.

**Table 2:**
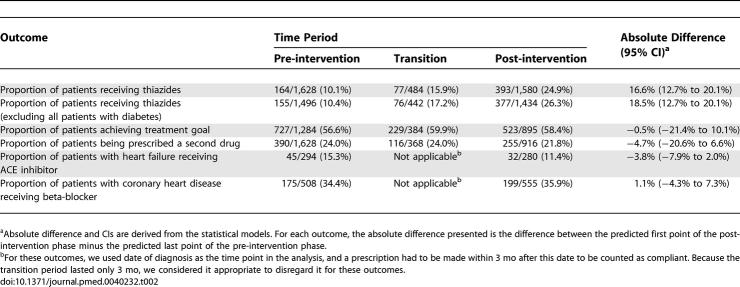
Main Findings, Aggregated per Time Period

**Figure 2:**
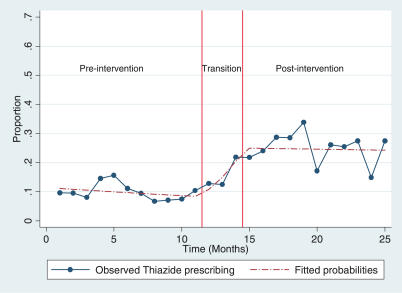
Proportion of Thiazide Prescriptions among All Prescriptions for Patients Started on Treatment for Uncomplicated Hypertension

During the baseline period, the proportion of thiazide prescriptions among all prescriptions made for patients started on treatment for uncomplicated hypertension was consistently around 10% (Figure 2). In the transition period, i.e., the 3 mo before the regulation went into effect, thiazide prescribing increased considerably and, after the new regulation was made operational, the proportions averaged around 25%. The new level of thiazide prescribing remained stable within the time frame of our study (Figure 2).

The results of the regression analysis for the main outcome are shown in Table 3. The regression coefficients can be interpreted as follows. During the baseline period, thiazide prescribing was decreasing by a statistically non-significant absolute rate of 0.3% per month (95% CI −0.2% to 0.8%). After the transition period, thiazide prescribing was nearly constant, decreasing by a statistically non-significant absolute rate of 0.05% per month (95% CI −0.65% to 0.35%). During the transition phase, the proportion of newly diagnosed patients who were prescribed thiazides increased by 4.9% per month (95% CI 0.3% to 9.1%). This corresponds to a 15% increase during the 3-mo transition phase. An alternative analysis excluding the transition phase confirms this. The odds ratio measuring the change in level from baseline to post-intervention is 3.7 (95% CI 2.5 to 5.6); using the predicted value for month 11, this can be interpreted as a change in level from baseline to post-intervention of 16.5% (95% CI 9.9% to 24.8%).

**Table 3:**
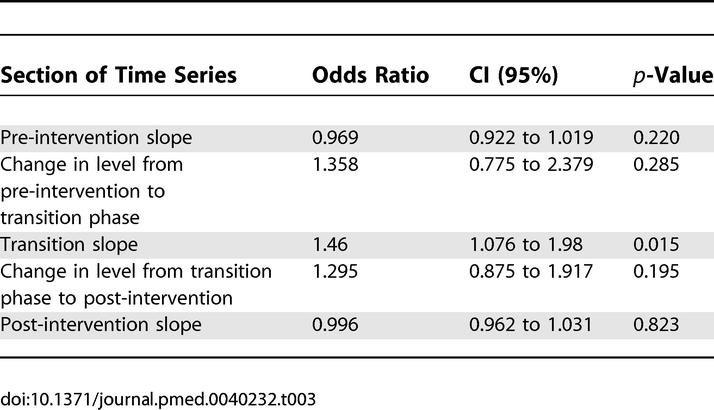
Regression Analysis for Main Outcome (Prescribing of Thiazides)

The results were similar when all patients with diabetes were excluded from the analysis (Table 2).

**Figure 3:**
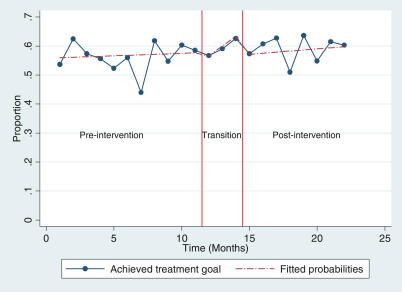
Proportion of Patients Achieving Treatment Goals (≤140/90 mm Hg) within 4 mo, among All Started on Treatment for Uncomplicated Hypertension

**Figure 4:**
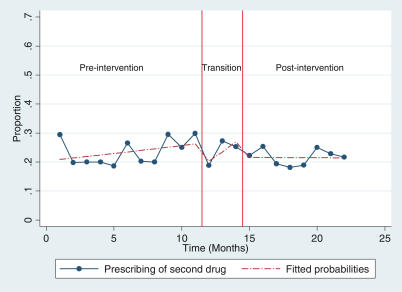
Proportion of Patients Receiving a Second Antihypertensive Drug within 4 mo of Starting Treatment for Uncomplicated Hypertension

The regulation did not appear to have an effect on the achievement of treatment goals or the need for adding or switching to other drug classes, with no statistically significant change over time (Figures 3 and 4). Also, for the use of ACE inhibitors for heart failure and the use of beta-blockers for coronary heart disease, we did not detect any statistically significant change (Table 2). However, the sample sizes for those analyses were much smaller.

**Figure 5:**
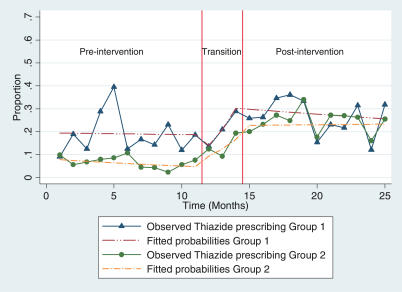
Proportion of Thiazide Prescriptions among Practices from Intervention Group in Earlier Trial (Group 1) and Other Practices (Group 2)

In the baseline period, the practices from the intervention group in the recently conducted trial were prescribing thiazides more frequently than the other two groups (absolute difference 12.3%; 95% CI 8.6% to 22.2%). This is in agreement with the results of the randomised trial [5]. As shown in Figure 5, the absolute increase in thiazide prescribing was also less pronounced in these practices compared to the ones that had not received the trial intervention. However, the difference was not statistically significant (absolute difference in intervention group practices 6.9%; 95% CI −6.0% to 15.7%).

We estimated drug expenditures during the pre-intervention period to be NOK3.7 million (€0.44 million, US$0.55 million). We estimated drug expenditures for the post-intervention period, after adjusting for differences in prescribing volumes, to be NOK3.5 million (€0.42 million, US$0.52 million). By extrapolating these figures to the national level, we estimated savings on drug expenditures to be NOK4.8 million (€0.58 million, US$0.72 million) over the first 12 mo post-intervention, which is equivalent to NOK1.06 per inhabitant (€0.13, US$0.16).

## Discussion

After the regulation was introduced making thiazides the only drugs qualifying for reimbursement for newly diagnosed uncomplicated hypertension, the rate of thiazide prescribing for this patient group increased from 10% to 25% in the practices included in our study. There was no apparent negative effect from the regulation in terms of achievement of treatment goals, the need for adding or switching to another drug class, or on the use of ACE inhibitors for heart failure or beta-blockers for coronary heart disease.

### Strengths and Weaknesses of the Study

The inherent weaknesses of an observational design make it difficult to establish a causal relationship between the intervention and the observed effects. However, the magnitude of change, the temporal relationship with the intervention, and the plausibility of a causal relationship make us confident that it exists.

We do not know how representative the study practices are of general practices in Norway. Almost 40% of the invited practices did not agree to participate in our study, and this may have introduced some degree of bias. It is also possible that the new rule had a different impact on physicians in and around Oslo than in the rest of the country. However, total sales of thiazides increased all over the country during the study period, although this increase is less in Oslo than in the rest of Norway (Table 4), which may mean that we have underestimated the effect size.

**Table 4:**
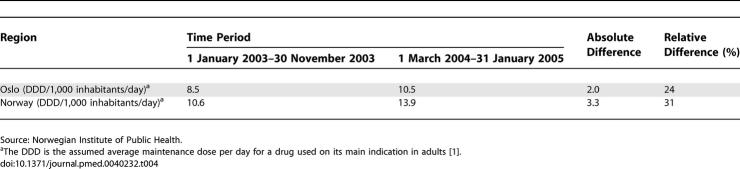
Total Sales of Thiazides (ATC Codes C03A and C03E) in Oslo and All of Norway

The price of many hypertensive drugs changed considerably in the year following the new regulation. While the increase in sales volume (defined daily dosages [DDDs]) for antihypertensive medications continued from 2003 to 2004, the total costs fell [1]. This was likely a result of specific measures unrelated to the new regulation, such as the “graded price model,” which ensures an automatic price reduction when a drug goes off patent [1]. For example, the price of many ACE inhibitors dropped substantially during the study period. However, it is also possible that the introduction of mandatory thiazide prescribing contributed to minor changes in drug prices, for instance the price increase that we observed for some thiazide preparations. The decrease in the price of non-thiazides, paralleled by an increase in thiazide prices, diluted savings associated with the regulation. We also conducted our economic analysis using 2003 prices for both study periods, and found that the estimated savings would have been approximately doubled without the observed price changes.

The bulk of savings resulting from the regulation can be expected over the following years, as an increasing number of patients will be started on thiazides. Assuming that prescribing patterns and drug prices remain unchanged from the post-intervention period and onwards, we estimate that the annual savings would be approximately NOK37 million, (€4.4 million, US$5.5 million) after 5 y, which is equivalent to NOK8.12 per inhabitant (€0.97, US$1.21) (discounting rate of 4%, in accordance with guidelines from the Norwegian Ministry of Finance). However, countermeasures by pharmaceutical companies can be anticipated and could influence prescribing patterns and drug costs, thus reducing the potential savings.

One-third of the included practices had a higher level of thiazide prescribing at baseline, reflecting their participation in a randomised trial of an intervention promoting the use of thiazides [5]. This may have led to a slight underestimation of the intervention effect. However, the estimated effect did not change significantly after exclusion of these practices from the analysis (an absolute increase from 15% to 17%).

In our opinion, the a priori specification of the transition period confers an advantage in our study. The estimated effect size of the intervention depends much on the choice of transition period. Without incorporating a transition period, the observed increase in thiazide prescribing over the final months before the new rule came into operation would have been included in the baseline period, making the effect size appear smaller. Conversely, if we had decided to extend the transition period by 1–2 mo in each direction, the difference before and after would have appeared to be greater than what we reported. This illustrates the potential for manipulation of time-series data and the need for detailed and publicly available protocols.

### Study Findings in Relation to the Overall Evidence

Earlier attempts at achieving changes in prescribing patterns have often been aimed at *influencing* professional behaviour, rather than *imposing* a change in behaviour. Various quality-improvement strategies have been employed, e.g., educational interventions, reminders, audit and feedback, or financial incentives. In a recent systematic review of randomised and non-randomised studies, the authors concluded that such strategies only lead to modest shifts in the prescribing patterns of antihypertensive medication (median absolute increase of 3%, interquartile range 1% to 5.5%) [10]. We recently conducted a randomised trial in Norwegian general practice evaluating the effect of a multifaceted intervention, including educational visits, audit and feedback, and computerised reminders [5]. The intervention led to an absolute increase in the rate of thiazide prescribing of 10%, which we considered a substantial effect compared to other interventions. However, the costs of implementing the intervention were also high, and the estimated savings were modest [11].

Our findings indicate that regulatory measures can be more effective in achieving changes in prescribing than conventional quality-improvement strategies. In addition, implementing a regulation such as the thiazide rule, with little monitoring and negligible penalties, makes it a far more cost-effective strategy. However, a problem with regulating physician behaviour is that it may be seen as infringing upon physician autonomy. The Norwegian Medical Association did not oppose the introduction of the new rule, but many critical voices were raised from the medical community, for example 19 consultants and professors at a university hospital in Oslo sent a letter of protest to the Minister of Health [12]. Other physicians argued that the medical profession has an ethical obligation to avoid unnecessary spending in order to make best use of the limited resources allocated to the health services.

The observed increase in the rate of thiazide prescribing was substantial in both absolute and relative terms (15% and 150%, respectively). On the other hand, even after the new regulation was introduced, non-thiazide drugs were being prescribed in 75% of all new cases of uncomplicated hypertension. It is highly unlikely that sound medical reasoning can explain this high degree of non-compliance among prescribing physicians. One possible cause is the existing controversy within the medical community regarding the appropriateness of thiazides as a first-line medication. Marketing of more expensive antihypertensives by pharmaceutical companies is likely to be an important factor [13]. Also, we cannot rule out the possibility of having included some patients who are not covered by the new regulation, e.g., patients with cardiovascular diseases who did not have their diagnosis coded in the medical record.

Restricting the number of drugs that are reimbursed is a widely adopted strategy to contain drug expenses. A major difference between the Norwegian policy and many analogous programmes is the opportunity that physicians have of exempting individual patients. Under the Norwegian regulation, the only requirement for reimbursement of other drugs is a note in the medical record, stating the reason why a thiazide could not be selected. Stricter conditions for granting exemptions are common in other settings, for example the need for approval before prescribing (prior authorisation) [14]. Limiting access to exemptions might have increased the effectiveness of the regulation, but would have entailed greater administrative costs, for example related to prior-authorisation procedures, and more resistance from stakeholder groups.

To our knowledge, there are only a very limited number of rigorous evaluations of the effect of introducing regulatory measures similar to the one we have carried out. Their effectiveness is unclear, although prior-authorisation schemes appear to have a substantial impact on prescribing [15]. Systematic reviews of the effects of pharmaceutical policies, including restrictions on reimbursed drugs, are underway [16].

Another approach to cost-containment for drugs is various forms of pricing policies. A recent systematic review included ten studies of the effect of reference pricing, i.e., a maximum level of reimbursement for a group of drugs assumed to be therapeutically equivalent [17]. The reviewers concluded that reference pricing can lead to a “shift in drug use towards less expensive drugs.” However, in a reference-pricing programme, the affected drugs typically belong to one drug class, e.g., ACE inhibitors. None of the included studies involved the use of a reference price for the full range of drugs used to treat a particular condition, thus making it difficult to compare the effects of reference pricing with the regulation that we have evaluated.

### Conclusions

There was a large increase in the use of thiazides for newly treated hypertension after a reimbursement rule was put into effect mandating the use of thiazides if there were no medical reasons for selecting other drugs. This increase occurred without monitoring adherence and without introducing penalties for non-adherence, and no changes were observed in the proportion of patients who achieved treatment goals or who were prescribed a second antihypertensive drug.

## Supporting Information

Alternative Language Abstract S1 Translation of the Abstract into Norwegian by Atle Fretheim (27 KB DOC)

Text S1 Study Protocol (94 KB PDF)

Text S2 Consent Form (in Norwegian) (94 KB DOC)

## Abbreviations

ACE: angiotensin-converting enzyme
ATC: Anatomic Therapeutic Chemical
CI: confidence interval
DDD: defined daily dosage
GEE: generalised estimating equations
ICPC: International Classification of Primary Care
ITS: interrupted time series
NOK: Norwegian kroner

## References

[pmed-0040232-b001] Rønning M (2006). Drug consumption in Norway 2001–2005.

[pmed-0040232-b002] Fretheim A, Aaserud M, Oxman AD (2003). The potential savings of using thiazides as the first choice antihypertensive drug: Cost-minimisation analysis. BMC Health Serv Res.

[pmed-0040232-b003] Vihovde MB (1999). Spørsmål til Helseministeren, Stortingets spørretime 24. November 1999.

[pmed-0040232-b004] Ramsay CR, Matowe L, Grilli R, Grimshaw JM, Thomas RE (2003). Interrupted time series designs in health technology assessment: Lessons from two systematic reviews of behavior change strategies. Int J Technol Assess Health Care.

[pmed-0040232-b005] Fretheim A, Oxman AD, Havelsrud K, Treweek S, Kristoffersen DT (2006). Rational prescribing in primary care (RaPP): A cluster randomized trial of a tailored intervention. PLoS Med.

[pmed-0040232-b006] Madsen S, Bryn E, Christensen HM (2004). Lavdose tiazid skal prøves først ved ukomplisert hypertensjon. Nytt om legemidler.

[pmed-0040232-b007] Mayor S (2005). Doctors urged to present views in an objective way to the media. BMJ.

[pmed-0040232-b008] Hafstad A (2003). Taper 200 mill. på dyre piller. Aftenposten.

[pmed-0040232-b009] Statistics Norway (2005). How much is it worth...?.

[pmed-0040232-b010] Walsh J, McDonald K, Shojania K, Sundaram V, Nayal S (2005). Hypertension care.

[pmed-0040232-b011] Fretheim A, Aaserud M, Oxman AD (2006). Rational prescribing in primary care (RaPP): Economic evaluation of an intervention to improve professional practice. PLoS Med.

[pmed-0040232-b012] Vollebæk LE (2004). Ullevål samlet mot blodtrykksvedtak. Dagens Medisin.

[pmed-0040232-b013] Wazana A (2000). Physicians and the pharmaceutical industry: Is a gift ever just a gift?. JAMA.

[pmed-0040232-b014] MacKinnon NJ, Kumar R (2001). Prior authorization programs: A critical review of the literature. J Manag Care Pharm.

[pmed-0040232-b015] Lexchin J (2002). Effects of restrictive formularies in the ambulatory care setting. Am J Manag Care.

[pmed-0040232-b016] Aaserud M, Dahlgren AT, Sturm H, Kosters JP, Hill S (2006). Pharmaceutical policies: Effects on rational drug use, an overview of 13 reviews (protocol). Cochrane Database Syst Rev.

[pmed-0040232-b017] Aaserud M, Dahlgren AT, Kosters JP, Oxman AD, Ramsay C (2006). Pharmaceutical policies: Effects of reference pricing, other pricing, and purchasing policies. Cochrane Database Syst Rev.

