# Supplementary material for: The Effects of Mandatory Prescribing of Thiazides for Newly Treated, Uncomplicated Hypertension: Interrupted Time-Series Analysis
Source: PLoS Med. 2007 Jul 10;4(7):e232. doi: 10.1371/journal.pmed.0040232 (PMC1904466; doi:10.1371/journal.pmed.0040232)
Supplement: Supplementary file 1 [file pmed.0040232.sd002.pdf]

Study protocol

Restrictions put on reimbursement of antihypertensive drugs other than thiazides: Interrupted time series

Atle Fretheim
Andy D Oxman
Norwegian Knowledge Centre for Health Services
PO Box 7004 St. Olavs plass
N-0130 Oslo
Norway

Abstract

Background

Antihypertensive medication is one of the major drug-expenditures for the national reimbursement scheme for drugs in Norway, adding up to nearly NKR 1.5 billion per year (€190 million), which is NKR 330 per inhabitant (€40). Norway is a country where the use of thiazides is particularly low and the use of expensive alternatives particularly high, and this has been an issue of concern. In 2004, the Norwegian Medicines Agency introduced a regulatory measure that obliged physicians to use thiazides as first-choice drugs for the treatment of uncomplicated hypertension, unless medical reasons suggested otherwise. The effect of this regulatory measure on prescribing habits is uncertain

Methods

We will conduct in interrupted time series analysis of prescribing of antihypertensive drugs to patients that are started on such medication in 60 practices in the Oslo-area in Norway. The primary outcome measure will be the proportion of non-thiazide prescriptions made for persons who are being started on medication for antihypertensive therapy.

Background

Antihypertensive medication is one of the major drug-expenditures for the national reimbursement scheme for drugs in Norway, adding up to nearly NKR 1.5 billion per year (€190 million) [1], which is NKR 330 per inhabitant (€40). An estimated 6-10% of the Norwegian population are being treated for hypertension [2].

There are several drugs to choose from for the treatment of hypertension, and in terms of effectiveness there is little or no difference between them [3,4]. One exception is low-doze thiazide-diuretics, which seem to protect better against developing heart failure than most other drug-classes [3,4]. From a public health and economic perspective the major difference between the drugs is undoubtedly the price. Many of the non-thiazide drugs cost tenfold of what thiazides do [2].

Norway is a country where the use of thiazides is particularly low and the use of expensive alternatives particularly high [2], and this has been an issue of concern for the Ministry of Health as well as Norwegian parliamentarians [5].

Late 2003 an agreement was reached at the political level to introduce regulations, which would restrict the reimbursement of antihypertensives other than thiazides. The Norwegian Medicines Agency was given the task of translating the decision into practice. They presented a new reimbursement rule Jan 15 2004, which was put into effect from March 1 2004.

Substantial savings are expected to result from the new reimbursement rule, however other factors than the anticipated increase in thiazide use may affect this outcome. For instance, some critics believe that thiazides are less effective in controlling blood pressure and that the need for add-on drugs will outweigh the economic gains.

The effects of health policy interventions at national or regional level are often poorly evaluated. Sometimes it is not feasible to conduct rigorous evaluations, such as a randomised controlled trial, due to pressure to implement quickly and widely. Sometimes the nature of the intervention is such that a control group is virtually impossible to establish, as with mass media campaigns, for example.

If a controlled designed evaluation is unfeasible, a before-and-after study may be an alternative. However, if measurements are only made once before and once after the intervention, the findings may be misleading. If, for instance, the prescribing of a drug was measured before and after an intervention and a difference was observed, this could simply reflect an ongoing trend, independent of the intervention. Another explanation for an observed change could be that events coinciding with the intervention had an influence on the prescribing of the drug. One way of increasing the robustness of a before-and-after analysis is to collect several data points before and after the intervention – an Interrupted time series (ITS) analysis.

We want to investigate whether changes in prescribing of thiazides for hypertension have occurred and whether the change can be attributed to the introduction of the new reimbursement rule. Also, we want to explore possible changes in proportions of people with blood pressure levels above recommended targets and the use of add-on drugs.

Methods

Design

Interrupted time series analysis, with 11 measurements at monthly intervals before and after the intervention. We will incorporate a transition period from December 2003 through February 2004.

Participants

60 General practices in the Oslo-area, Norway. The sample will be drawn from three different practice populations. 20 practices will be drawn from the practices that were randomised to the intervention group in trial we recently conducted that addressed prescribing of antihypertensive drugs [6]. 20 other practices will be drawn from the control group in the same trial. Finally, 20 practices will we drawn among the practices that did not participate in the trial.

Intervention

A new rule from the Norwegian Medicines Agency, which only allowed for reimbursement of thiazides for patients started on medication for uncomplicated hypertension, in this case

defined as not having hypertensive organ damage, gout, reduced glucose tolerance or untreated diabetes. If there were medical reasons not to choose a thiazide other drugs would be reimbursed, on the condition that rational for this was noted in the medical record.

Information about the new rule was circulated to all physicians through the January edition of the bulletin of the Norwegian Medicines Agency [7]. The rule was also widely debated in the media.

No specific sanctions against non-compliant physicians were announced, but it was said that the National Insurance Administration, which manages the reimbursement of drugs, were about to increase their monitoring of adherence to reimbursement rules.

The intervention was not independent of other changes over time. For instance, the new regulation prompted a debate, widely covered in the media, where opinion-leaders and other stakeholders had strongly divergent views regarding the use of thiazides for the treatment of hypertension.

There is no reason to believe that the intervention may have affected data-collection.

Data-collection

We will collect data from the practice medical records. The data will include all prescribed antihypertensive drugs from three years before and one year after the reimbursement rule was implemented.

For all patients that have been given a prescription for an antihypertensive drug, we will also collect information on cardiovascular diagnoses (ICPC-codes K74-80, K84, K99) and prescribing of cardiovascular drugs (ATC-code C01). This is to identify patients who may be taking the drugs for other reasons than hypertension. We will also extract blood-pressure measurements, and only patients with a recorded measurement above 140/90 will be included in the main analyses.

Because antihypertensive drugs are also prescribed to treat migraine or hyperthyroidism, we will identify patients with these diagnoses (N89, T85) and prescriptions for drugs to treat

these conditions (N02C, H03B). If these diagnoses or prescriptions are recorded the patient will be excluded from the analysis.

The new reimbursement rule only includes patients treated for uncomplicated hypertension, defined in this case as not having target-organ damage, goat, decreased glucose tolerance or untreated diabetes. In order to identify patients with these conditions we will extract information on relevant diagnoses (K87, T92, T89, T90) and prescriptions (M04, A10). We will not attempt to identify patients with decreased glucose tolerance, as we do not believe that the data we extract will enable us to do so in a reliable way. Patients with a recorded glucose measurement ≥ 7 mmol/l and no recorded prescriptions for antidiabetic medication or other diabetes-aids, will be considered as having untreated diabetes, and thus excluded from the main analyses.

Outcome measures

The primary outcome measure will be the proportion of non-thiazide prescriptions made for persons who are being started on medication for antihypertensive therapy. We will conduct a subgroup analysis where all diabetes-patients are excluded (patients with untreated diabetes are excluded from the main analysis). We will also conduct subgroup analyses comparing the three groups of practices that participate in the study.

In addition, the following outcomes will be measured:

- The proportion of patients that have not reached recommended blood pressure goals among all that started treatment in the year before vs. the year after the intervention.
- The proportion of patients started on a second antihypertensive drug year before vs. the year after the intervention.
- The proportion of patients with a diagnosis of heart failure not being prescribed an ACE-inhibitor <u>plus</u> the proportion of patients with diagnosis of coronary heart disease not being prescribed a beta-blocking agent, year before vs. the year after the intervention. (Proxy measure for inappropriate prescribing).

These are objective outcomes, thus no blinding or reliability testing of outcome evaluation is considered necessary.

Ethics

We will ask for consent from all the participating practices. Physicians and patients will not be identifiable. No ethical approval is needed. We will apply the Norwegian Data Inspectorate for approval for handling of the data.

Sample size and statistics

Based on data from a study we have conducted [6] we estimate that an average general practitioner starts blood pressure lowering therapy on 10 patients per year. In the same study the average number of physicians per practice was 2.7. Thus, we can expect 20-30 new patients per practice per year. In order to detect a 25% relative reduction, with a power 80% and a statistical significance level of 5%, on the prescribing of non-thiazide drugs for hypertension, a sample of 60 practices should suffice, according to expert advice (Craig R. Ramsey, University of Aberdeen).

Competing interests

Both authors are employed by the Norwegian government, which has substantial interest in containing the costs of health care.

Authors' contributions

ADO developed the idea for the study, with contributions from AF. AF drafted the study protocol and ADO made critical revisions to it.

Acknowledgements

The study will be funded by the Norwegian Ministry of Health, which will have no influence on the choice of methods or conduct of the study.

Reference List

1. Drug consumption in Norway 1999 - 2003. 2004. Oslo, The Norwegian Institute of Public Health.

2. Fretheim A, Aaserud M, Oxman AD: **The potential savings of using thiazides as the first choice antihypertensive drug: cost-minimisation analysis.** *BMC Health Serv Res* 2003, **3:** 18.

3. Blood Pressure Lowering Treatment Trialists' Collaboration: **Effects of different blood-pressure-lowering regimens on major cardiovascular events: results of prospectively-designed overviews of randomised trials.** *Lancet* 2003, **362:** 1527-1535.

4. Psaty BM, Lumley T, Furberg CD, Schellenbaum G, Pahor M, Alderman MH *et al*.: **Health outcomes associated with various antihypertensive therapies used as first-line agents: a network meta-analysis.** *JAMA* 2003, **289:** 2534-2544.

5. Spørsmål til Helseministeren, Stortingets spørretime 24. november 1999. www.stortinget.no/spti/dw-o1999112405-021.html . 10-2-2003.

6. Fretheim A, Oxman AD, Treweek S, Bjorndal A: **Rational Prescribing in Primary Care (RaPP-trial). A randomised trial of a tailored intervention to improve prescribing of antihypertensive and cholesterol-lowering drugs in general practice [ISRCTN48751230].** *BMC Health Serv Res* 2003, **3:** 5.

7. Madsen S, Bryn E, Christensen HM: **Lavdose tiazid skal prøves først ved ukomplisert hypertensjon.** *Nytt om legemidler* 2004, **27:** 7-8.
