# Supplementary material for: The Effects of Mandatory Prescribing of Thiazides for Newly Treated, Uncomplicated Hypertension: Interrupted Time-Series Analysis
Source: PLoS Med. 2007 Jul 10;4(7):e232. doi: 10.1371/journal.pmed.0040232 (PMC1904466; doi:10.1371/journal.pmed.0040232)
Supplement: Supplementary file 2 [file pmed.0040232.sd001.doc]

# Sammendrag

Bakgrunn

Å evaluere effektene av et nytt refusjonsvilkår for blodtrykksmedikamenter. Det nye vilkåret innebar at tiazider skulle benyttes som førstehåndspreparat ved oppstart av behandling ved ukomplisert hypertensjon. Målet med det nye refusjonsvilkåret var å begrense medikamentutgiftene.

Metoder og funn

Vi gjennomførte en avbrutt tidsserieanalyse basert på forskrivningsdata fra før og etter innføringen av det nye refusjonsvilkåret. Alle pasienter som begynte med blodtrykkssenkende behandling ved 61 allmennlegekontor i Norge inngikk i analysen. Refusjonsregelen ble foreslått av Helsedepartementet og godkjent av Stortinget. Etterlevelse av regelen ble nesten ikke overvåket og det var ingen sanksjoner mot leger som ikke forskrev i tråd med det nye refusjonsvilkåret. Vårt primære resultatmål var andelen av tiazidforskrivninger blant alle forskrivninger som ble gjort ved oppstart av blodtrykkssenkende behandling. Blant de sekundære resultatmålene var andelen av pasienter som oppnådde anbefalte behandlingsmål innen fire måneder etter behandlingsstart, og andelen pasienter som innen fire måneder ikke hadde begynte med et annet blodtrykkssenkende preparat. Vi sammenliknet også legemiddelkostnader før og etter innføringen av refusjonsvilkåret. I perioden før vilkåret ble innført ble tiazider forskrevet til 10 % av pasientene som begynte på blodtrykkssenkende behandling. Denne andelen økte jevnt i overgangsperioden for å stabilisere seg rundt 25 %. Vi fant ingen statistisk signifikante forskjeller for andre resultatmål. Oppnåelse av behandlingsmål var noe høyere etter innføringen av refusjonsvilkåret (56.6 % vs. 58.4 %), og forskrivningen av et legemiddel nummer to gikk litt ned (24.0 % vs 21.8 %). Medikamentutgiftene gikk ned med anslagsvis 4.8 million kroner (€ 0.58 millioner, $US 0.72 millioner) i løpet av det første året, som tilsvarer 1.06 kroner per innbygger (€ 0.13, $US 0.16).

Konklusjoner

Forskrivningen av tiazider for ukomplisert hypertensjon ble mer enn doblet etter at forskrivning av tiazider ble innført som vilkår for refusjon av legemiddelutgifter. Innsparingene som følge av det nye refusjonsvilkåret ble likevel moderate. Det var ingen signifikant endringer i oppnåelse av behandlingsmål eller i forskrivninger av et annet blodtrykkssenkende medikament blant dem som begynte på behandling.
