# Supplementary material for: The Effects of Mandatory Prescribing of Thiazides for Newly Treated, Uncomplicated Hypertension: Interrupted Time-Series Analysis
Source: PLoS Med. 2007 Jul 10;4(7):e232. doi: 10.1371/journal.pmed.0040232 (PMC1904466; doi:10.1371/journal.pmed.0040232)
Supplement: Supplementary file 3 [file pmed.0040232.sd003.doc]

Vår ref.: 2005/294, Prosj. 263

Samtykkeerklæring for deltakelse i ”Tiazidregelevalueringen”

- Vi er kjent med målsetting for prosjektet
- Vi er klar over at det er frivillig å delta i prosjektet
- Vi vet at deltakelse i prosjektet innebærer at det ved hjelp av et dataprogram hentes ut anonymiserte data fra journalsystemet på legekontoret
- Vi har fått tilfredsstillende svar på våre eventuelle spørsmål om prosjektet
- Vi er klar over at vi når som helst kan trekke oss fra prosjektet, uten nærmere begrunnelse

Jeg samtykker i å delta i prosjektet på vegne av

___________________________________________________________

Navn på legekontor

____________________________________________________________

Legekontorets adresse

Lege: ___________________________________	Dato___________

## Returner per post til:

Nasjonalt kunnskapssenter for helsetjenesten

v/Kari Håvelsrud

Postboks 7004, St. Olavs plass

0130 Oslo

***Eller per faks:***

23 25 50 40
